# New proposal of viral genome representation applied in the classification of SARS-CoV-2 with deep learning

**DOI:** 10.1186/s12859-023-05188-1

**Published:** 2023-03-11

**Authors:** Luísa C. de Souza, Karolayne S. Azevedo, Jackson G. de Souza, Raquel de M. Barbosa, Marcelo A. C. Fernandes

**Affiliations:** 1 Laboratory of Machine Learning and Intelligent Instrumentation, Federal University of Rio Grande do Norte, Natal, RN 59078-970 Brazil; 2 Department of Pharmacy and Pharmaceutical Technology, University of Granada, Granada, Spain; 3 Department of Computer Engineering and Automation, Federal University of Rio Grande do Norte, Natal, RN 59078-970 Brazil; 4 Bioinformatics Multidisciplinary Environment (BioME), Federal University of Rio Grande do Norte, Natal, RN 59078-970 Brazil

**Keywords:** COVID-19, SARS-CoV-2, GSP, CGR DFT, Deep learning

## Abstract

**Background:**

In December 2019, the first case of COVID-19 was described in Wuhan, China, and by July 2022, there were already 540 million confirmed cases. Due to the rapid spread of the virus, the scientific community has made efforts to develop techniques for the viral classification of SARS-CoV-2.

**Results:**

In this context, we developed a new proposal for gene sequence representation with Genomic Signal Processing techniques for the work presented in this paper. First, we applied the mapping approach to samples of six viral species of the Coronaviridae family, which belongs SARS-CoV-2 Virus. We then used the sequence downsized obtained by the method proposed in a deep learning architecture for viral classification, achieving an accuracy of 98.35%, 99.08%, and 99.69% for the 64, 128, and 256 sizes of the viral signatures, respectively, and obtaining 99.95% precision for the vectors with size 256.

**Conclusions:**

The classification results obtained, in comparison to the results produced using other state-of-the-art representation techniques, demonstrate that the proposed mapping can provide a satisfactory performance result with low computational memory and processing time costs.

## Introduction

The World Health Organization (WHO) declared, on January 30, 2020, that the COVID-19 outbreak, a disease caused by the Severe Acute Respiratory Syndrome Coronavirus 2 (SARS-CoV-2) virus, constituted a Public Health Emergency of International Concern (PHEIC), given the rapid spread of the virus, in such a way that two weeks after the first diagnosed case, other thousand patients tested positive for Coronavirus [1, 2]. In July of 2022, the total number of reported cases of the disease surpassed the 540 million mark, with 6.3 million deaths caused by the virus. Because of the high spread rate associated with this disease, it is vitally important to diagnose the infected patients so that they are properly treated and isolated to avoid contagion to other individuals.

In this scenario, the standard test adopted to perform the diagnosis based on the extraction of viral RNA is the Quantitative Reverse Transcription Polymerase Chain Reaction (qRT-PCR) [3]. However, the work presented in [4] found a false-negative rate of about $26.7\%$ and $27\%$ for critical and moderate cases, respectively. The study presented in [4] analyzed 866 samples of the qRT-PCR (from the respiratory tracts) of 213 patients infected with the Coronavirus. All samples were collected from 0 to 7 days after the onset of the disease. This false-negative result is believed to be due to a specific RNA virus mutation, where SARS-CoV-2 has an average evolution rate of approximately $10^{-4}$ substituted nucleotide per year [5].

In this context, the classification, description, and comparison of viral sequences based on their genomic characteristics can help study phylogenetic relationships and mechanisms of action of pathogens, contributing to the development of vaccines and other prophylaxis measures [6]. Thus, it is essential to improve techniques for analyzing and classifying the viral genome, and in bioinformatics, this analysis is performed using two main methods. The first method is about techniques that use sequence alignments, such as BLAST [7] and BLAT [8]. Such algorithms look for matches of bases or groups of bases in the same order in two or more sequences. However, the disadvantages of such methods are the high computational cost required, which limits their use in large genomic databases [9], in addition to assuming that cDNA (complementary DNA) sequences are linearly arranged, which is not the case for viral sequences. Furthermore, the application of such methods is not suitable in scenarios where the sequences present significant divergences or in comparing sequences with millions of nucleotides [10–12]. The second method encompasses techniques in which sequence alignment is not performed (free-alignment) [13]. This method was developed as an alternative for solving biological problems where alignment techniques have limitations. It has been applied in several studies, such as the analysis of the evolution of organisms and regulatory sequences as promoters and inhibitors, the identification of cis-regulatory modules (CRM), and the comparison of sequences using data from next-generation sequencing technologies [11].

Free-alignment techniques can be divided into two main categories. The first is based on word frequency and works by creating count vectors of pattern occurrences in sequences, then applying quantization metrics of similarity between sequences. The second category includes techniques that do not depend on the resolution of the sequences. Instead, based on information theory, they seek to identify, focusing only on the representation of the sequences, the information shared between the analyzed genomic data [13, 14]. Some of these techniques are based on the characteristics of genomic sequences, and such methods include the use of machine learning (ML) to classify viral sequences. This classification occurs in two stages, the first can be characterized as a mapping of biological sequences in a feature space, and the second stage consists of processing the data by an ML technique [15, 16].

DNA holds genetic information in its molecules that systematise living organisms’ development and functioning and viruses. Techniques for mapping or representing DNA sequences, or cDNA, transform nucleotides into numerical information [17]. Numeric representations of genetic sequences can be divided into three categories: single value mapping, in which each nucleotide will be associated with a unique value in one-dimensional space; multidimensional sequence mapping, where each nitrogenous base will be replaced by a vector containing a point in multidimensional space; and cumulative mapping, where a random walk model will accumulate the contribution of consecutive values associated with the nucleotides to form a curve [18].

Genomic Signal Processing is based on the use of theory, algorithms, and mathematical digital signal processing methods for the analysis, processing and use of genomic data [15, 17–19]. GSP techniques can identify hidden periodicity and distribution properties. Therefore, the use of these tools in conjunction with numerical representations of DNA sequences can provide more information about the genetic profile of organisms, compared to conventional representation methods [17]. The proposal presented in [18] used GSP techniques to convert nucleotide sequences to a graphical representation to be used in classifying three types of functional genomes performed by a deep learning architecture. The work proposed in [20] developed a new form of numerical mapping of DNA sequences using a multidimensional representation associated with the Discrete Fourier Transform (DFT), one of the most consolidated and applied GSP tools, due to its ability to transform genetic sequences into the frequency domain to reveal features not displayed in the time domain. In the work presented in [21], GSP techniques for feature selection were used, together with machine learning methods, to develop an automatic classification system for SARS-CoV-2, SARS-CoV and MERS-CoV.

The use of machine learning based on deep neural networks has shown significant results in viral classification. The technique proposed in [16] uses a deep convolutional neural network (CNN) to perform viral classification, applying the method to dengue, HIV-1, influenza A, hepatitis B and C, and depending on the viral type and the number of associated subtypes, obtained an F1-score from 0.85 to 1.0. In turn, the work presented in [22] made use of a convolutional neural network based on text classification models to classify DNA sequences represented by one-hot encoding vectors. The method was tested in 12 datasets, with the average accuracy ranging from $88.99\%$ to $99.06\%$, depending on the dataset. In the research carried out in [23], ViraMiner was developed, a viral identification method that contains two branches of CNNs designed to detect frequency patterns in metagenomic contigs, for contigs with 300 bp, the method achieved 0.923 for the area under the receiver operating characteristic (ROC) curve.

However, given the complexity of interpreting genomic sequences, which deal with large amounts of data, the performance of the machine learning techniques is directly associated with how the sequences are represented [24]. This way, this work aims to develop a new strategy for representing viral cDNA sequences, such as SARS-CoV-2, using a set of genomic signal processing techniques. The new strategy uses a pipeline of Chaos Game Representation (CGR) associated with Discrete Fourier Transform to be used in deep learning methods for viral classification. Such representation of genetic sequences generates a new viral signature containing the information in a new feature space that is considerably shorter in length than the original genomic sequence. This new representation can decrease the memory required for data handling, enabling the use of large amounts of genomic sequences in machine learning analyses. Consequently, the time cost required for viral classification is significantly lower, not exceeding 17 s per fold, for training the proposed network architecture. The main contributions of this paper are the following:

- We propose a methodology for representing viral sequences with GSP tools to generate reduced viral signatures.
- We used the proposed methodology to classify the SARS-CoV-2 virus in a dataset containing samples from the same virus family and help discriminate SARS-CoV-2, which is strongly related to other coronavirus species.
- In the viral classification, we use Deep Learning architectures, which present performance and implementation similar or superior to conventional machine learning techniques.
- We showed that the classifier could differentiate between species with high accuracy even with only 64 to 256 values in the viral signature vector.
- We compared the representation performance with techniques consolidated in the literature and showed that the proposed approach presents similar or superior performance.

## Representation proposal

**Fig. 1:**
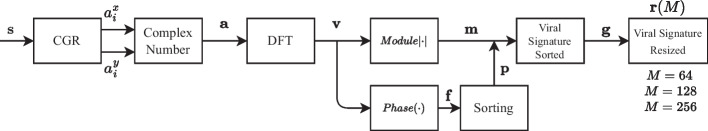
Proposal sequence representation scheme. Where $\textbf{s}$ represents a cDNA sequence applied in the proposed technique, $a_i^x$, $a_i^y$, $\textbf{a}$, $\textbf{v}$, $\textbf{m}$, $\textbf{f}$, $\textbf{p}$, $\textbf{g}$ and $\textbf{r}$ are vectors obtained after each operation mentioned inside the boxes and *M* represent the final sizes of the viral signature

Figure 1 illustrates the proposed representation technique, in which a sequence of cDNA of length *N* is expressed as

1 $$\begin{aligned} \textbf{s} = [s_1,\dots ,s_i,\dots ,s_N] \end{aligned}$$

where each *i*-th element $s_i$ represents one of the possible nucleotides of the cDNA sequence, i.e., $s_i \in \{\textrm{A}, \textrm{C}, \textrm{T}, \textrm{G}\}$. The proposal uses two techniques of processing genomic signals in cascade, aiming to create a unique signature for each *i*-th cDNA sequence. The processing techniques are CGR and DFT, which will be detailed in future subsections [25, 26].

### Dataset

For this study, each $\textbf{s}$, associated with one of the $12\text {,}467$ viral genome sequence samples from 67 countries, were downloaded through the National Genomics Data Center (NGDC) database. All downloaded viral sequences are complete, have high-coverage and have N’s number less than $0.01\%$. The dataset contains samples from six species: Severe Acute Respiratory Syndrome-related Coronavirus (SARS-CoV-2); Betacoronavirus 1; Middle East Respiratory Syndrome-related Coronavirus (MERS-CoV); Human Coronavirus NL63 (HCoV NL63); Human Coronavirus 229E (HCoV 229E); and Human Coronavirus HKU1 (HCoV HKU1). Belonging to the Coronaviridae family, from the kingdom Riboviria, they have a genome length ranging from $26\text {,}000$ to $32\text {,}000$ bp. The sequences formed by nucleotide bases are presented as character vectors, where each letter represents a specific nucleotide, guanine (G), adenine (A), thymine (T), and cytosine (C). Table 1 presents a summary of the data from the samples used in this work.

**Table 1:** Samples of viral sequences

Viral species	Sequence information		
	Num. of seq.	Seq. len.	Seq. len.
		min. (*N*)	max. (*N*)
SARS-Cov-2	11,969	26,973	30,018
Betacoronarivus 1	140	30,536	31,029
MERS-CoV	258	29,267	30,150
HCoV NL63	55	27,302	27,832
HCoV 229E	27	26,592	27,307
HCoV HKU1	18	29,367	29,983

In the developed method, the viral signatures were classified into two classes. The first one has all $11\text {,}969$ SARS-CoV-2 samples, containing only the original strain, which means, that no Coronavirus variant is present in the dataset. The other class has all the other virus species in the dataset combined, resulting in 498 sequences.

### Chaos game representation (CGR)

Proposed in [27], the CGR is a methodology capable of providing numerical and graphical representations of genetic sequences through iterative function systems (IFSs) [20, 27]. The CGR maps the cDNA sequence characterized by the vector $\textbf{s}$ (see Eq. 1) into a two-dimensional space through the symbols $a_n^x$ and $a_n^y$, expressed as

2 $$\begin{aligned} a_n^x = \frac{1}{2}s_n^x +\frac{1}{2}a_{n-1}^x, \text { for } n=1,\dots ,N \end{aligned}$$

and

3 $$\begin{aligned} a_n^y = \frac{1}{2}s_n^y +\frac{1}{2}a_{n-1}^y, \text { for } n=1,\dots ,N \end{aligned}$$

where

4 $$\begin{aligned} s_n^x = {\left\{ \begin{array}{ll} 1 &{} \text {if } s_n=\textrm{A} \\ -1 &{} \text {if }s_n=\textrm{T} \\ -1 &{} \text {if }s_n=\textrm{C} \\ 1 &{} \text {if }s_n=\textrm{G} \\ \end{array}\right. } \end{aligned}$$

and

5 $$\begin{aligned} s_n^y = {\left\{ \begin{array}{ll} 1 &{} \text {if } s_n=\textrm{A} \\ 1 &{} \text {if }s_n=\textrm{T} \\ -1 &{} \text {if }s_n=\textrm{C} \\ -1 &{} \text {if }s_n=\textrm{G} \\ \end{array}\right. }. \end{aligned}$$

In the proposed technique, the initial condition is assumed as ($n=0$), $a_0^x=0$ and $a_0^y=0$ [20, 25]. Thus, each base associated with a $s_n$, will represent a point in the two-dimensional space containing the coordinates $a_n^x$ and $a_n^y$, and these values will be related to a complex number in the form $a_n^x+ ja_n^y$, resulting in the vector $\textbf{a}$, expressed as

6 $$\begin{aligned} \textbf{a} = \left[ a_1^x + ja_1^y, a_2^x + ja_2^y,\dots , a_N^x + ja_N^y \right] . \end{aligned}$$

**Fig. 2:**
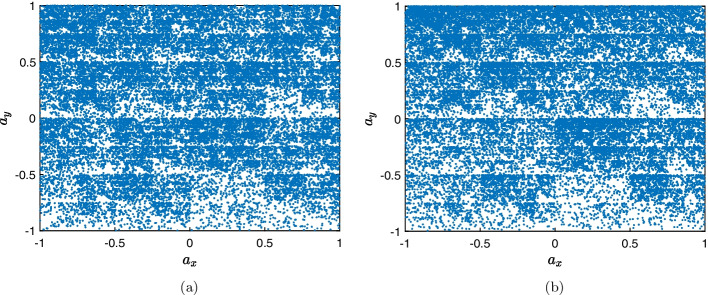
Example of viral representation using CGR, where each point in the image is the mapping of a nucleotide. **a** SARS-CoV-2 Virus (GU553363). **b** Betacoronavirus-1 Virus (KX538977)

Figure 2 illustrates two examples of viruses from the Coronaviridae family, mapped with CGR, in which it is observed that each virus holds a distinct signature.

As shown in Fig. 1, in the next stage of the representation proposal, the vector $\textbf{a}$ will be used in the DFT.

### DFT and vector sorting

Based on the works presented in [20, 26], this proposal makes use of DFT, aiming to generate a signature in the frequency domain of the genomic signal, given that from the analysis of the spectrum provided, periodicities and latent information of the sequences of nucleotides can be observed more easily than in time domain analyses [26, 28].

As illustrated in Fig. 1, the vector of complex numbers $\textbf{a}$ of length *N* passes through a DFT generating the vector $\textbf{v}$, which can be expressed as

7 $$\begin{aligned} \textbf{v} = \left[ v_1, v_2,\dots , v_N \right] \end{aligned}$$

where each *i*-th element $v_i$ can be defined as

8 $$\begin{aligned} v_i = \sum _{n=0}^{N-1}v_n e^{-j \frac{2 \pi }{N}in}. \end{aligned}$$

After calculating the DFT, because its data are in complex form, it is necessary to decompose the modulus and phase components of the vector $\textbf{v}$, generating the vectors $\textbf{m}$ and $\textbf{f}$, respectively [29]. The vector $\textbf{m}$ can be expressed as

9 $$\begin{aligned} \textbf{m} = \left[ m_1, m_2,\dots , m_N \right] \end{aligned}$$

where each *i*-th element $m_i$ is the amplitude at a given frequency an can be expressed as

10 $$\begin{aligned} m_i = |v_i| \end{aligned}$$

The DFT phase, represented by the vector $\textbf{f}$, is presented as

11 $$\begin{aligned} \textbf{f} = \left[ f_1, f_2,\dots , f_N \right] \end{aligned}$$

where each *i*-th element $f_i$ is the phase of the distributed transform from $-\pi$ to $\pi$ being expressed as

12 $$\begin{aligned} f_i =\angle v_i. \end{aligned}$$

**Fig. 3:**
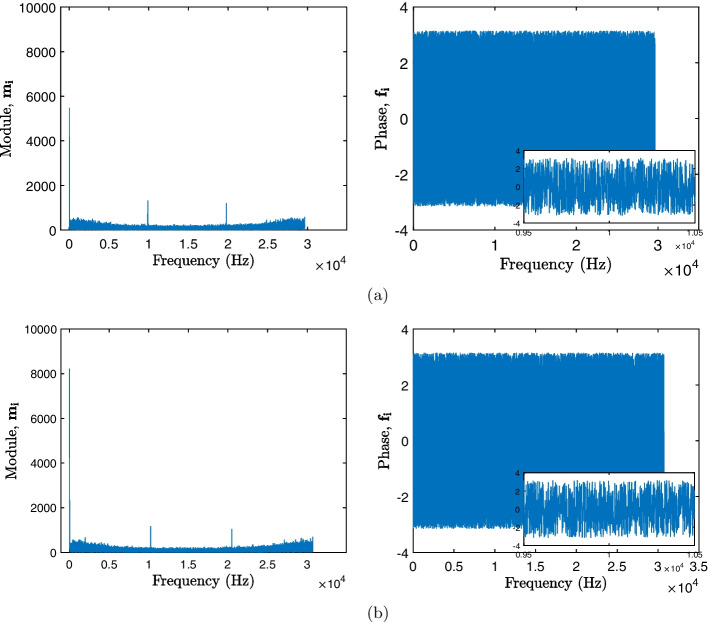
DFT module and phase response of viral samples. The left image presents the module response $\mathbf {m_i}$ for each virus, the right image illustrates the transform phase $\mathbf {f_i}$, and inside the little window in this image shows a zoom in of the phase between $0.95 \times 10^4$ Hz and $1.05 \times 10^4$ Hz. **a** SARS-CoV-2 Virus (GU553363). **b** Betacoronavirus-1 Virus (KX538977)

Figure 3 shows the DFT of two viral samples obtained from the CGR as shown previously in Fig. 2, where the first image of Fig. 3a, b presents the transform module ($\textbf{m}$), and the second panels of the images shows the phase ($\textbf{f}$).

As seen in Fig. 3, response in module signatures, similar viruses have similar maximum frequency values, but in different phases. Therefore, an increasing sorting of the vector $\textbf{f}$ is performed, resulting in a vector of positions of the ordering $\textbf{p}$, represented as

13 $$\begin{aligned} \textbf{p} = \left[ p_1, p_2,\dots , p_N \right] \end{aligned}$$

and these positions are used to sort the vector module $\textbf{m}$, resulting in a new vector $\textbf{g}$, expressed as

14 $$\begin{aligned} \textbf{g} = \left[ g_1, g_2,\dots , g_N \right] \end{aligned}$$

where each *i*-th element $g_i$ will be the amplitude value ordered according to its phase position, as seen

15 $$\begin{aligned} g_i = m_{p_i}. \end{aligned}$$

**Fig. 4:**
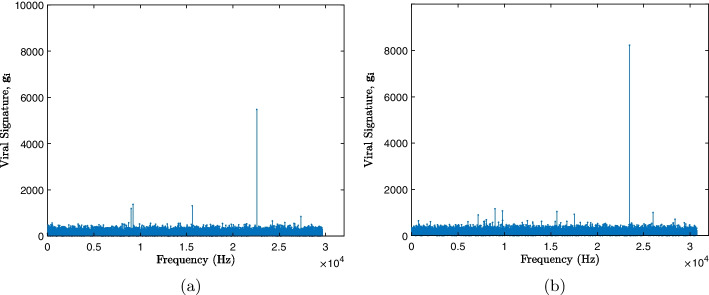
Ordered viral signatures. **a** SARS-CoV-2 Virus (GU553363). **b** Betacoronavirus-1 Virus (KX538977)

From the ordering of the vectors, a new vector $\textbf{g}$ was obtained with the same modulus function as the original, but with different positions, relative to the function of its phases, thus increasing the differentiation between signatures of similar viruses as is displayed in Fig. 4, which shows the new ordered viral signature of the samples presented in Fig. 3.

The works [20, 30–32] present strategies similar to those of the present study, applying the CGR and then calculating the DFT of the genetic sequences, however, the proposals do not make use of the DFT phase ordinate. The techniques developed by [20, 30] use the transform power spectrum, while [31] chose to use only the amplitude spectrum together with the Pearson correlation coefficient. In the proposal presented in [32], the average values of the smoothed DFT were calculated. Thus, it is important to highlight that not using phase information can disregard the location of local maximum frequency values, focusing only on their amplitude value. In Fig. 3, it is possible to observe that the two viral samples have frequency maxima around the values 0 Hz, $1 \times 10^4$ Hz and $2 \times 10^4$ Hz, and that around of $\ 1 \times 10^4$ Hz and $\ 2 \times 10^4$ Hz, the amplitude for the two samples is similar, however, observing the phase around the frequency value $\ 1 \times 10^ 4$Hz, as shown in the image amplification in the right quadrant of Fig. 3, the two samples present different phase profiles. As seen in Fig. 4, after sorting, the highest frequency values are no longer in similar positions.

### Length reduction

Given that the ordered viral signature vector, $\textbf{g}$, have different lengths, as observed in Table 1, which presents the minimum and maximum values for *N*, and that due to the use of the DFT, the amount of relevant information is associated with a small number of maximum frequency values [28], a reduction in the data size was carried out until the vectors have the lengths 64 and 128 per signature. These length values were chosen after experimenting with different sizes, as in the classification carried out by a CNN, they presented better results in the characterization of genetic data.

For this purpose, we selected the *M* highest values of $\textbf{g}$, where *M* assumes 64 or 128, generating the vector $\textbf{b}$ and their positions in the original vector, which form the vector $\textbf{o}$, presented as

16 $$\begin{aligned} \textbf{b} = \left[ b_1, b_2,\dots , b_M \right] \end{aligned}$$

e

17 $$\begin{aligned} \textbf{o} = \left[ o_1, o_2,\dots , o_M \right] . \end{aligned}$$

The vector of positions $\textbf{o}$ was then ordered in ascending order and, similar to the ordering of the transform performed in the previous section on the vector $\textbf{m}$, the new positions were used in the highest modulus values presented in vector $\textbf{b}$, getting the vector with reduced dimension $\textbf{r}$ with size *M*, expressed as

18 $$\begin{aligned} \textbf{r} = \left[ r_1, r_2,\dots , r_M \right] \end{aligned}$$

where each element $r_i$ was given by

19 $$\begin{aligned} r_i = b_{o_i(ordered)}. \end{aligned}$$

**Fig. 5:**
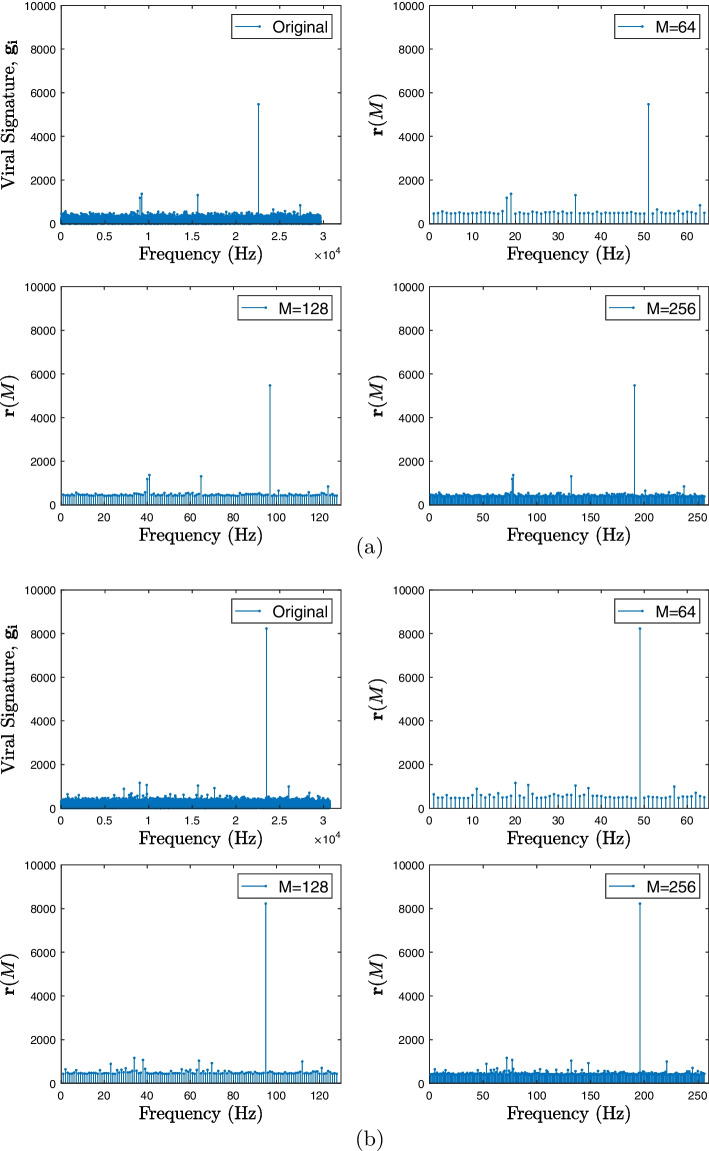
Size reduction of viral samples for all *M*. The first image shows the original viral signature $\mathbf {g_i}$, and the second presents the resized vector $\textbf{r}(M)$ for $M=64$. The third image shows the resized vector $\textbf{r}(M)$ for $M=128$. Finally, the last image shows the resized vector $\textbf{r}(M)$ for $M=256$. **a** SARS-CoV-2 Virus (GU553363). **b** Betacoronavirus-1 Virus (KX538977)

In this way, each point of $\textbf{r}$ will be in position relative to the other maximum values of the original sequence $\textbf{g}$. For example, Fig. 5 shows the result of the compression of two viral samples for all sizes of *M*.

After the viral signatures reduction of the length of the vector, the technique of representation of the cDNA sequences is finished, with this representation being then able to be analyzed by deep learning techniques.

## Deep neural network architecture

**Fig. 6:**
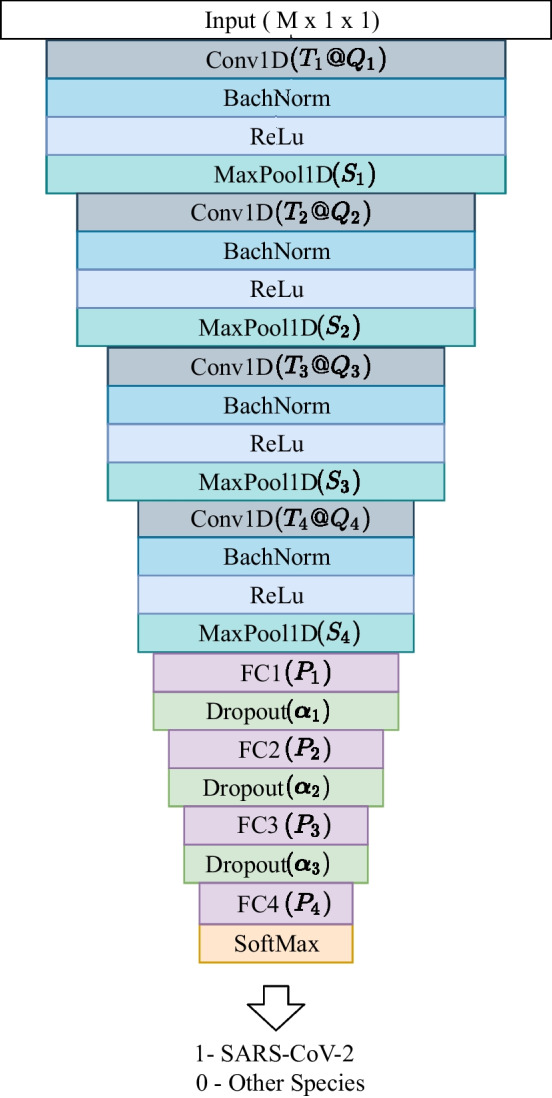
Convolutional Neural Network architecture used for classification of SARS-CoV-2

Following the literature proposals [15, 16, 33], this work employed genomic signal processing techniques to represent the genetic sequences together with a convolutional neural network (CNN) to classify them into two classes: SARS-CoV-2 or other species. The architecture of the Deep Neural Network used is a one-dimensional convolutional network model, where the length of the viral signatures influenced the choice of some parameters, such as the input size, the number of layers, and the size of the filters. The classifiers provided discrete outputs characterized by the values 1 and 0. Figure 6 present the proposed model architecture for the viral classification of SARS-CoV-2, where $M\times 1\times 1$ is the input dimension, $T_n$ is the filter size of the *n*-th layer (convolutional layer), $Q_n$ is the number of filters of the *n*-th convolutional layer, $S_n$ is the pool size of the *n*-th max pool layer, $P_n$ is the number of neurons in the *n*-th fully connected layer, and $\alpha _n$ is the dropout percentage of the *n*-th dropout layer. The CNN proposed model architecture was designed with 25 layers with an input layer, four convolutional layers represented by Conv1D($T_n@Q_n$) where $n=1,\dots ,4$, four batch normalization layers, four activation function layers represented by ReLu, four max pool layers represented by MaxPool1D($S_n$) where $n=1,\dots ,4$, four fully connected layers represented by FC($P_n$) where $n=1,\dots ,4$, four dropout layers represented by Dropout($\alpha _n$) where $n=1,\dots ,4$, and a softmax layer with output layer.

Several training and validation tests were carried out to reach the final CNN architecture. Initially, to define the size associated with convolution filter parameters, i.e., Conv1D($T_n@Q_n$) where $n=1,\dots ,4$, four strategies were assembled, and the one with the best validation accuracy (ACC) was chosen. The parameters used for each strategy are presented in Table 2, and the ACC results are presented in Table 3 for each *M* value.

**Table 2:** Convolutional filter parameters used in strategy tests

Strategy 1	Strategy 2	Strategy 3	Strategy 4
$T_1=8$ and $Q_1=16$	$T_1=4$ and $Q_1=16$	$T_1=8$ and $Q_1=8$	$T_1=4$ and $Q_1=8$
$T_2=4$ and $Q_2=8$	$T_2=4$ and $Q_2=8$	$T_2=4$ and $Q_2=8$	$T_2=4$ and $Q_2=8$
$T_3=2$ and $Q_3=2$	$T_3=2$ and $Q_3=2$	$T_3=2$ and $Q_3=2$	$T_3=2$ and $Q_3=2$
$T_4=2$ and $Q_4=2$	$T_4=2$ and $Q_4=2$	$T_4=2$ and $Q_4=2$	$T_4=2$ and $Q_4=2$

**Table 3:** ACC results for different convolutional filter strategy tests

Input size layer (*M*)	Strategy 1 (%)	Strategy 2 (%)	Strategy 3 (%)	Strategy 4 (%)
64	83.62	97.64	97.62	96.23
128	97.00	97.00	90.38	93.40
256	96.75	98.05	94.50	95.12

From the values presented in the Table 3, it was possible to observe that for all *M* sizes, strategy 2 offered the best accuracy values, being this the one chosen for the proposed architecture. The following parameter analyzed was the pool size of the all max pool layer, i.e., MaxPool1D($S_n$) where $n=1,\dots ,4$. As in the initial architecture, four layers of a one-dimensional max pool were used. The last two layers $S_3$ and $S_4$ needed to present a size equal to 2, given that the minimum input size limited the downsampling performance. Then, in Table 4, the ACC values are presented for three pool size values, where only the values of the first two max pool layers were modified, MaxPool1D($S_1$) and MaxPool1D($S_2$), keeping the last two with pool length equal to 2.

**Table 4:** Validation accuracy of different pool size for MaxPool1D($S_1$) and MaxPool1D($S_2$) layers

Input size layer (*M*)	$S_1=S_2=2$ (%)	$S_1=S_2=4$ (%)	$S_1=S_2=8$ (%)
64	93.25	97.00	97.50
128	89.00	90.62	96.88
256	87.75	96.75	92.25

Again, the three values of *M* presented higher validation accuracy values for the same configuration, with pool size set to 8 for $S_1$ and $S_2$, which was subsequently chosen for the final architecture. The last parameter analyzed was the size of FC layers. For FC($P_1$), FC($P_2$), and FC($P_3$) layers were made two strategies. In strategy one, it was used $P_1=64$, $P_2=128$, and $P_3=256$. In the other direction, the second strategy it was used $P_1=256$, $P_2=128$, and $P_3=64$. Table 5 presents the results associated with FC layers tests.

**Table 5:** ACC for FC($P_1$), FC($P_2$), and FC($P_3$) layers

Input size layer (*M*)	Strategy 1	Strategy 2
	$P_1=64$, $P_2=128$, and $P_3=256$ (%)	$P_1=256$, $P_2=128$, and $P_3=64$ (%)
64	98.00	98.60
128	98.12	97.38
256	98.80	99.12

It was verified from the Table 5 that the strategy two ($P_1=256$, $P_2=128$, and $P_3=64$) showed better accuracy for $M=64$ and $M=256$, so this was selected. From the information obtained in the experiments detailed above, the final architecture is shown in Table 6.

**Table 6:** Final parameters of the Convolutional Neural Network architecture

Layer	Description	Values
	Input	
1	($M \times 1 \times 1$)	$M=64$, 128 or 256
2	Conv1D	$T_1=4$ and $Q_1=16$
3	BachNorm	−
4	ReLu	−
5	MaxPool1D	$S_1=8$
6	Conv1D	$T_2=4$ and $Q_2=8$
7	BachNorm	−
8	ReLu	−
9	MaxPool1D	$S_2=8$
10	Conv1D	$T_3=2$ and $Q_3=2$
11	BachNorm	−
12	ReLu	−
13	MaxPool1D	$S_3=2$
14	Conv1D	$T_4=2$ and $Q_4=2$
15	BachNorm	−
16	ReLu	−
17	MaxPool1D	$S_4=2$
18	FC1	$P_1=256$
19	Dropout	$\alpha _1=0.6$
20	FC2	$P_2=128$
21	Dropout	$\alpha _2=0.6$
22	FC3	$P_3=64$
23	Dropout	$\alpha _3=0.6$
24	FC4	$P_4=2$
25	SoftMax	2 classes

## Results

The algorithms of this work were implemented in Matlab 2020 (License: 596681) on a computer with the configurations: Intel Core i5-7200U with 2.50 GHz CPU and 8 GB RAM. As shown in section Dataset, the number of examples in the class ”Other Species” is 498. To balance the data and avoid bias, 400 cDNA sequences were selected for training and repeated five times, resulting in a set with 2000 samples. To gather the test set, the network selected all remaining samples of SARS-Cov-2 and 98 of the other unknown viruses. To evaluate the convolutional neural network model, *k*-fold cross-validation with $k=5$ was used.

The network was trained during 50 epochs and used the RMSProp optimizer with a learning rate of 0.001 to minimize the loss function, which was the Cross-entropy function. Furthermore, the batch size chosen for the network training was equal to 512. Therefore, the time needed to process the representation was 0.006 seconds for each cDNA sequence, and the training lasted about 12 seconds (in mean) for $M=64$, 14 seconds for $M=128$ and 17 seconds for the size $M=256$, per fold.

**Fig. 7:**
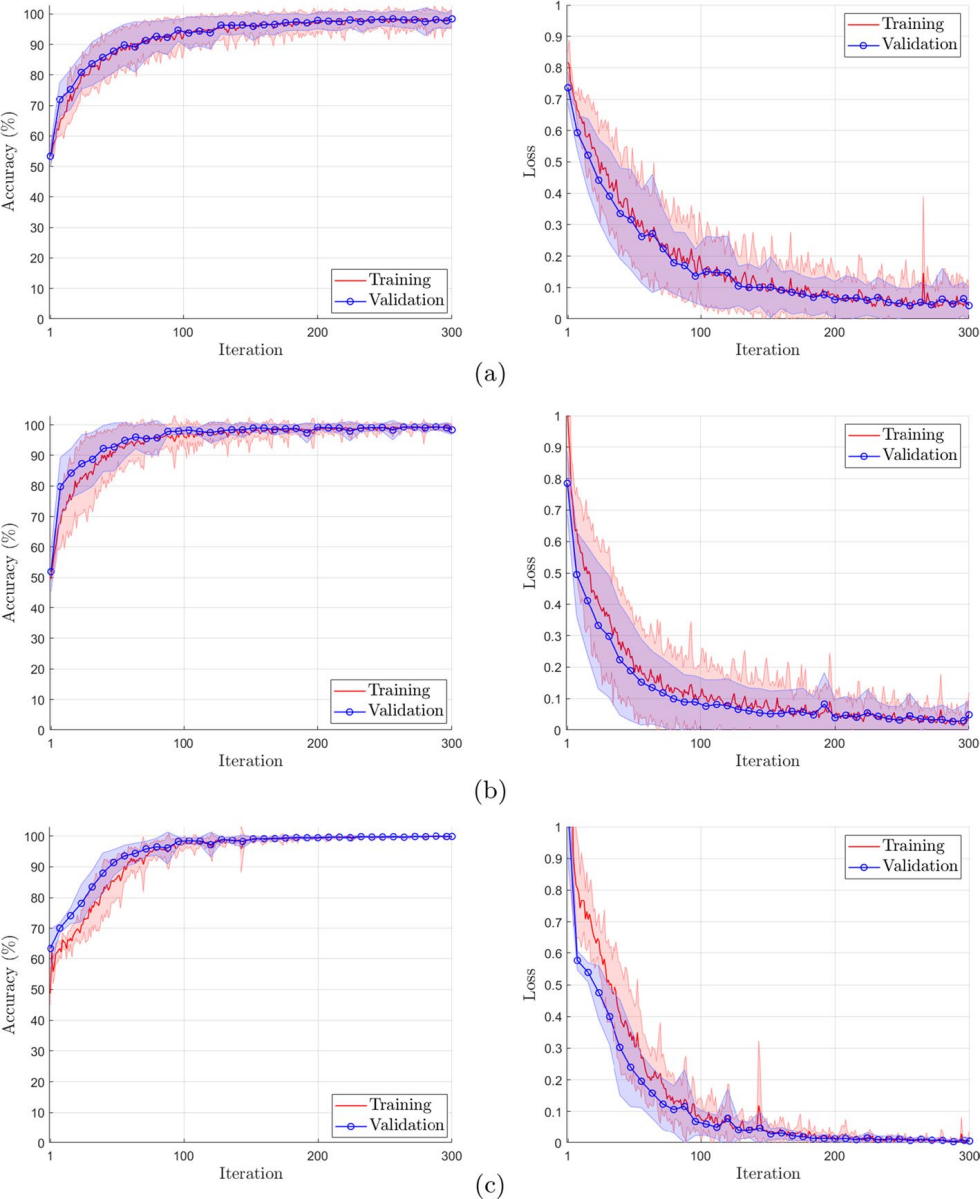
Learning curve plot for accuracy(left) and loss(right) of the proposed model for the different sizes of *M*. **a** $M=64$; **b** $M=128$; **c** $M=256$

Besides cross-validation, three dropout layers were added to the final architecture, a technique that randomly ignores units and their connections during model training to avoid overfitting and improve the performance of the neural network. Figure 7 shows the average curves with a standard deviation of the accuracy and loss for training and validation of the model. The presented in Fig. 7 show that the model does not suffer from overfitting (high variance) or underfitting (high bias). The reduced difference between the training and validation curves consolidates the absence of overfitting.

After training, the network was tested, and the performance of the deep learning network in the classification of the COVID-19 virus was analyzed using the representation with the ordered phase. Table 7 presents the performance measures accuracy (ACC), sensitivity (SEN), specificity (SPE), precision (PRE), F1-score, and AUC. This metric will evaluate the training effects of the classifier for the dataset so that the higher the AUC value, the better its performance [34].

The ROC curve is a graph that presents the performance of a classifier, being produced by plotting on the *y* axis the true positive rate, that is, the sensitivity performance metric. On the *x* axis, the false positive rate, which represents $1 - \text {specificity}$, for the test values [35]. From this, the area under the curve (AUC) of the ROC curve performance metric was obtained.

**Table 7:** Comparison of the performance of the Learning Network rating for the dimension sizes $M=64$, $M=128$, and $M=256$

*M*	Performance metrics					
	ACC (%)	SEN (%)	SPE (%)	PRE (%)	F1-Score (%)	AUC
64	98.35	98.40	93.06	99.93	99.16	0.9834
128	99.08	99.12	93.88	99.94	99.53	0.9911
256	99.69	99.74	94.69	99.95	99.84	0.9950

The method was also validated by comparing genomic sequence representation techniques already consolidated in the literature. For this, the same samples were used for training, validation, and testing, in addition to the proposed architecture, adopting the same parameters. Table 8 presents the results of the comparison in terms of the average processing time required to apply the representation in only one sequence, memory required to store $1\text {,}000$ viral signatures obtained by mappings, DNN training time for the set of $3\text {,}200$ samples, in addition to the performance metrics already exposed in Table 7.

**Table 8:** Comparison of the performance of the proposed method with other representations in the literature

Performance	ASCII	EIIP	One-hot	This work		
Metrics	[36]	[37]	Encoding [38]	$M=64$	$M=128$	$M=256$
Processing time p/sequence	0.0180 s	0.0181 s	0.648 s	0.0063 s	0.0063 s	0.0064 s
Memory required p/1000 vectors	8.86 MB	16.8 MB	16.9 MB	471 KB	941 KB	1.83 MB
Training time per fold	48.4 min	46.55 min	54.5 min	12 s	14 s	17 s
ACC	$98\%$	$98.5\%$	$96\%$	$98.35\%$	$99.08\%$	$99.69\%$
SEN	$96.1\%$	$98\%$	$98\%$	$98.40\%$	$99.13\%$	$99.74\%$
SPE	$100\%$	$99\%$	$94.2\%$	$93.06\%$	$93.88\%$	$94.69\%$
PRE	$100\%$	$99\%$	$94.2\%$	$99.93\%$	$99.94\%$	$99.95\%$
F1-score	$98.01\%$	$98.49\%$	$96.06\%$	$99.16\%$	$99.53\%$	$99.84\%$

The work presented in [21] used the EIIP representation. Already in [16] applied ASCII in the cDNA sequences for viral classification. The approaches presented [22, 23] employ one-hot encoding to classify proteins and viruses, respectively. Observing Table 8, it’s possible to conclude that the proposed representation of sequences presents similar or superior performance to the consolidated techniques but with a lower computational cost and time.

Finally, it was conducted a performance comparison of COVID-19 virus classification algorithms available in the scientific literature, based on Machine Learning and Deep Learning, with the method proposed in the present work with the best performing *M*, as seen in Table 9.

**Table 9:** Comparison of the performance of SARS-CoV-2 classification algorithms

Reference	Methodology	ACC	SEN	SPE	PRE	F1-Score
Arslan and Arslan [39]	CpG based features, KNN	$98.4\%$	$99.2\%$	–	$98.4\%$	$98.8\%$
Singh et al. [40]	Three-base periodicity, Random Forest	$97.47\%$	$96.19\%$	$98.25\%$	–	–
Randhawa et. al. [41]	k-mers, six supervised learning models.	$100\%$	–	–	–	–
Lopez-Rincon et at. [7]	Primer design, CNN.	$98.73\%$	–	$100\%$	–	–
This work	GSP, CNN.	$99.69\%$	$99.74\%$	$94.69\%$	$99.95\%$	$99.84\%$

To perform the comparisons in the Table 9, only papers based on genome sequence analysis of the SARS-CoV-2 virus were selected. The dataset used by Arslan and Arslan [39] included the same species present in this work, based on the features extraction from the CpG island, obtaining a sensitivity of $99.2\%$. However, the method proposed in the present work had greater values for all performance metrics analyzed. Singh et al. [40] used data without any pre-processing to select 8 biomarkers to replace the need for whole genome analysis, reducing the processing consumption of the classifiers. However, their method obtained the lowest accuracy among the algorithms exposed in Table 9, the low number of samples of viral sequences pointed out as one of the limitations of the work. The same limitation of the research by Randhawa et al. [41] since they used only 29 SARS-CoV-2 sequences. Moreover, the result of $100\%$ of accuracy obtained in the classification may be due to factors such as overfitting caused by the small number of samples in the dataset. As Lopez-Rincon et at. [7] that besides having a dataset with few samples it was still an unbalanced dataset but reached a specificity of $100\%$, which can mean that the primer sets developed did not present any false positive result.

## Discussion

The machine learning performance is directly associated with how genomic data chains, which deal with voluminous amounts of data, are mapped to a new feature space [24]. It is possible to observe this relationship between sequence representation and classifier performance with the results obtained by the DNN developed to detect SARS-CoV-2. Where it’s evident an increase, even if tenuous, of the values of the adopted metrics with the growth of the *M* value so that $M=256$ presented superior results of accuracy, sensitivity, specificity, precision, F1-score, and AUC, since it presented fewer false-positive and false-negative results.

The information obtained by DFT reflects the periodicities and distributions of the nitrogenous bases in the sequences. As the proposed representation method selects only the largest *M* values of the modulus after DFT transform, it can be assumed that the 256 vector size potentially presents more intrinsic features for each species, making it easier for DNN to classify them. However, considering that all signature sizes showed AUC greater than 0.900, it can be concluded that the proposed method can represent cDNA sequences even after significant size reduction so that the length of the final vectors obtained by the mapping is less than $1\%$ of the original sequence size of the Coronaviridae family viruses. In addition, it was also found that all false-positive results obtained in the classification were viral sequences belonging to the genus Betacoronavirus containing the SARS-CoV-2 virus. And all samples from the genus Alphacoronavirus were classified correctly.

Furthermore, from the Tables 8 and 9 analyses, we can see the classifier efficiency compared to other algorithms for the detection of COVID-19. In the 8 table, six representations of cDNA sequences methods were tested to the same supervised learning model. And remarkably, the computational and time consumption for the three values of *M* is lower than for other techniques, without significant differences in the performance metrics, so the accuracies for $M=128$ and 256 were the highest presented in the table. Similarly, compared to other works that performed the classification of SARS-CoV-2 with machine learning techniques, seen in Table 9, the results obtained in the present work have similar or superior performance, including the papers that used a resembling dataset to the one used in this work.

However, the low number of samples in the dataset was one of the limitations encountered in the research development, especially for the other species class, which was counteracted by data balancing tactics. Even so, it is essential to use more data to testify to the effectiveness of the representation method. Another difficulty identified was the limitation of the biological interpretation of the results provided by the DNN since it’s impossible to know the rules used to generalize the data, generating a Black Box effect of the model.

## Conclusion

The pandemic caused by the spread of the SARS-CoV-2 virus significantly impacted the health and economic scenery worldwide. Thus, studying its phylogenetic characteristics and evolutionary behavior is of utmost importance in combating viral proliferation. So, in this work, a new representation of cDNA sequences was proposed, based on the use of genomic signal processing techniques, applied to viral sequences of the Coronaviridae family for the classification of the COVID-19 virus, and later, applied to the analysis of variants of the SARS-CoV-2 virus. Initially, CGR was applied to the genomic sequences, obtaining spatial coordinates and applying to DFT. Compared to other works that used Fourier transform in preprocessing genetic data samples, the present method uses the phase information in combination with the amplitude information of the signals to increase the sample’s differentiation.

The size reduction of the viral signature vectors allows a viral classification with low computational cost, both in the training time of the classification model and in the amount of memory required for storage, relevant characteristics in the treatment of large amounts of data, as is the case of genomic sequences available by next-generation sequencing technologies. Despite the low processing cost, the method had no performance loss, reaching an accuracy of $98.35\%$, $99.08\%$ and $99.69\%$, and AUC of 0.9834, 0.9911, and 0.9950 for vector length equal to 64, 128, and 256, in the classification, performed with SARS-CoV-2 viruses and other species from the same family, such as Betacoronarivirus 1, MERS-CoV, HCoV NL63, HCoV 229E, and HCoV HKU1.

## Data Availability

The datasets and code generated and/or analysed during the current study are available in the Mendeley Data repository, DOI: 10.17632/kbxsjgkxfp.1

## Abbreviations

ACC: Accuracy
AUC: Area under curve
cDNA: Complementary DNA)
CGR: Chaos game representation
CNN: Convolutional neural network
DFT: Discrete Fourier transform
DNN: Deep neural network
GSP: Genomic signal processing
ML: Machine learning
PRE: Precision
qRT-PCR: Quantitative reverse transcription polymerase chain reaction
ROC: Receiver operating characteristic
SARS-CoV-2: Severe acute respiratory syndrome coronavirus 2
SEN: Sensivity
SPE: Specifity
